# Three-dimensional printing versus traditional surgery for inveterate pelvic and acetabular fractures: A retrospective study of 37 patients

**DOI:** 10.1097/MD.0000000000036149

**Published:** 2023-11-17

**Authors:** Fulin Tao, Lin Li, Dawei Wang, Jinlei Dong, Dongsheng Zhou, Wenhao Song

**Affiliations:** a Department of Orthopedic Surgery, Shandong Provincial Hospital Affiliated to Shandong First Medical University, Jinan, Shandong, China; b Department of Orthopedic Surgery, Shandong Provincial Hospital, Cheeloo College of Medicine, Shandong University, Jinan, Shandong, China; c Department of Orthopedic Surgery, Tengzhou Central People’s Hospital Affiliated to Jining Medical University, Tengzhou, Shandong, China.

**Keywords:** inveterate fracture, pelvic and acetabular fracture, three-dimensional printing

## Abstract

Treatment of deformed pelvic and acetabular fractures is a considerable challenge for orthopedic surgeons. The aim of this study was to assess the availability of a three-dimensional (3D) printing model used in patients with inveterate pelvic and acetabular fractures by comparing 3D printing technology with conventional surgery. We conducted a retrospective review of patients with inveterate pelvic and acetabular fractures treated in our department between January 2008 and June 2020. The patients were divided into 2 groups according to their willingness. Perioperative data and clinical outcomes were compared to evaluate clinical efficacy. The *t*-test, Fisher exact test, and multivariable logistic regression analysis were conducted. A *P* value of .05 or less was considered to be statistically significant (two-tailed). Thirty-seven patients were enrolled in our study. Seventeen patients were divided into the case group treated by 3D printing model-assisted preoperative planning, and 20 patients were divided into the control group treated by conventional surgery. Patients treated with the 3D printing model had significantly shorter operation times, less blood loss, and shorter fluoroscopy times. Patients in the case group also showed better pain relief according to visual analog scale scores. However, the elevations in pelvis and hip joint functional outcomes were similar between the 2 groups, and no significant difference was shown in the radiological result. The usage of 3D printing techniques in patients with inveterate pelvic and acetabular fractures is of great importance in preoperative preparation and optimization of surgery but cannot improve postoperative function compared with conventional treatment.

## 1. Introduction

Pelvic fractures account for 1% to 3% of all types of fractures^[1]^ with an incidence of 16.1%^[2]^ when they are complicated with acetabular fractures. These injuries are usually caused by high-kinetic energy forces, and the common mechanisms include motor vehicle accidents and falling from a height.^[3]^ The patients usually arrive at the emergency department with unstable hemodynamics and multiple organ injuries. Inveterate pelvic and acetabular fractures usually refer to fractures with a course of more than 3 weeks^[2]^ and are usually due to delayed surgery or misdiagnosis on account of the urgency of maintaining stable vital signs and limited insight provided by X-plains. The porosis and sophisticated anatomy of the pelvis and acetabulum make it difficult to achieve anatomical reduction. Common plain X-ray and computed tomography (CT) images provide limited insight into the physical configuration of fracture components, especially for complex acetabular fractures.^[4]^ Accurate osteotomy and deformity correction are significant challenges, even for experienced orthopedic surgeons. The treatment of inveterate pelvic and acetabular fractures usually involves extended operation time and increased blood loss compared with the treatment of normal fractures and leads to unsatisfactory results.

Advances in the three-dimensional (3D) printing technique provide surgeons with detailed models showing the precise anatomical morphology of fracture configurations.^[5]^ In this study, we used 3D printing to help improve the accuracy of osteotomy and prepare pre-contoured fixation plates according to fracture segments. Three-dimensional printing techniques have been presented in complex orthopedic surgery including that of the spine, pelvis, and joints.^[6]^ Previous studies have demonstrated that it is an effective and reliable method for treating anterior pelvic ring and acetabular fractures^[4,5,7–9]^ and have shown an advantage for elective surgery in cases of severe acetabular bone loss.^[10]^ However, the use of 3D printing techniques in the preoperative planning of inveterate pelvic and acetabular fractures, to our knowledge, has not been reported previously. We hypothesized that a 3D printing technique would optimize the surgery process and improve the clinical outcomes. The aim of this study was to assess the availability of 3D printing used in patients with inveterate pelvic and acetabular fractures compared with conventional radiographic planning.

## 2. Materials and methods

We analyzed the data of all patients with inveterate pelvic and acetabular fractures treated in our department between January 2008 and June 2020. The generic surgical indications were the presence of clinical signs including neurological pain, limb discrepancy, and vertical or rotational displacement of the pelvic ring observed on radiography. The inclusion criteria were (1) injury time > 3 weeks, (2) undergoing open reduction and internal fixation, (3) undergoing only one surgery, and (4) 18 years < age < 60 years. The exclusion criteria were (1) injury time < 3 weeks, (2) undergoing conservative treatment, (3) fractures of tumorous or osteoporotic bone. The patients were divided into 2 groups according to their willingness. Patients in the case group underwent 3D printing model-assisted preoperative planning. Patients in the control group underwent conventional surgery assisted only by plain X-rays and CT radiographs. Permission for this study was obtained from the Medical Ethics Committee at the authors’ institution, and the patients and/or their families were informed that data from the research would be submitted for publication, and gave their consent.

### 2.1. Surgical procedures

All surgeries were performed under general anesthesia by one surgeon.

**Figure 1.**
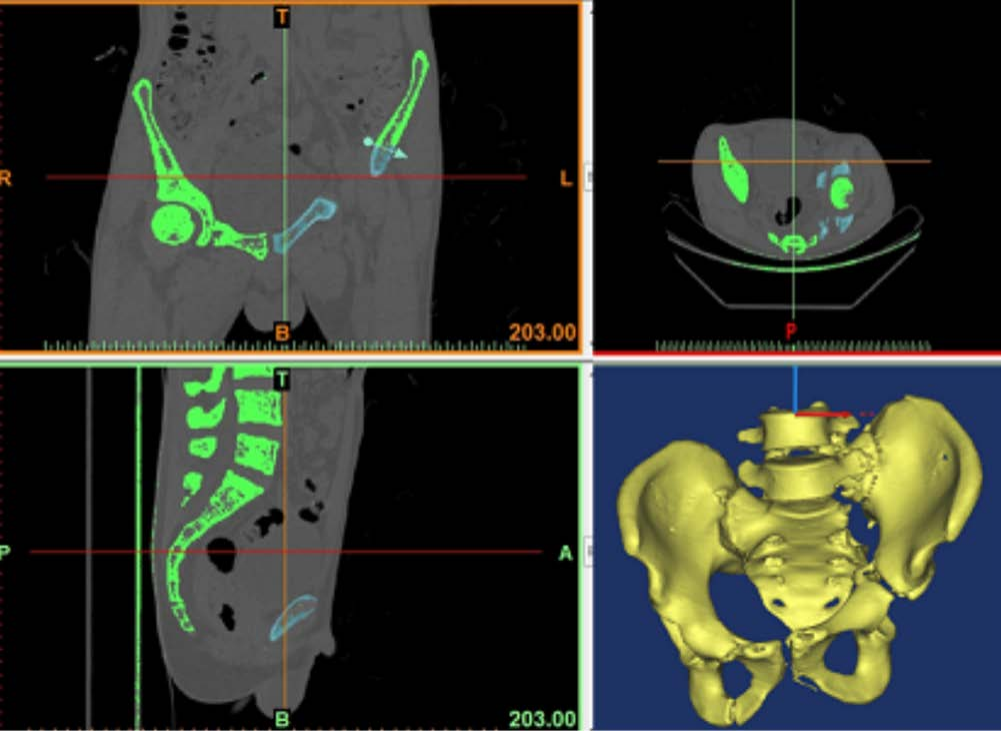
Three-dimensional reconstruction of the malformed pelvis.

**Figure 2.**
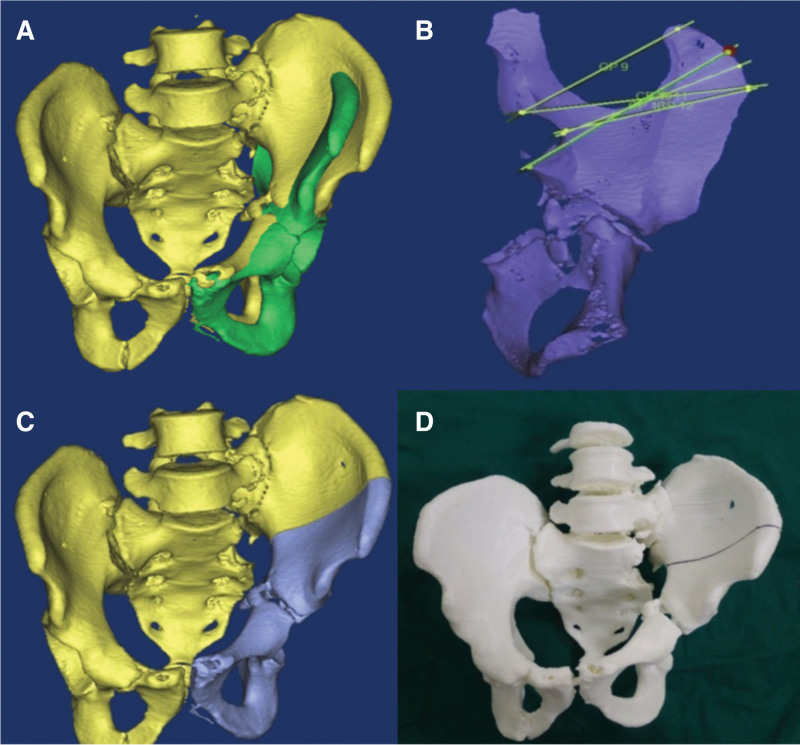
(A and B) A schematic design of visual osteotomy. (C and D) Final decision of the osteotomy plane.

**Figure 3.**
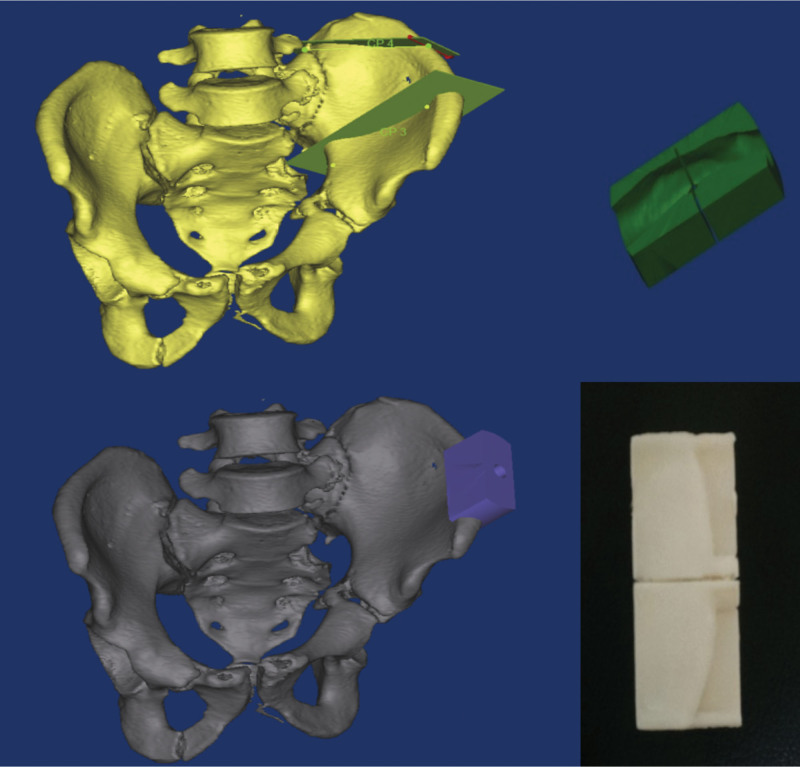
Design of the surgery guide for osteotomy.

**Figure 4.**
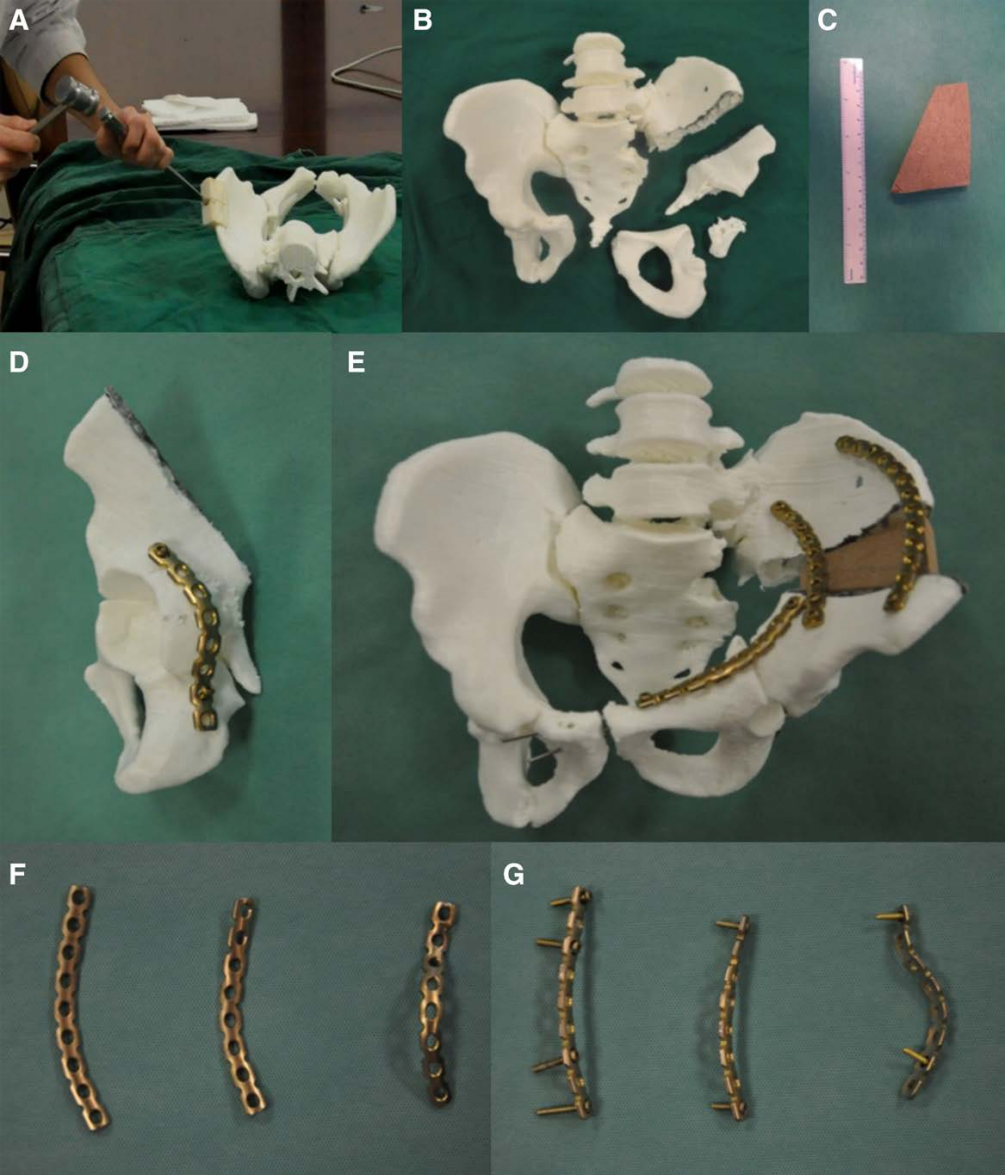
(A and B) Surgical simulation of osteotomy on a model based on a 3D-printed surgery guide. (C) Bone graft module based on osteotomy. (D) Fixation of the posterior wall of the acetabulum. (E) Anterior view of the final simulated surgery on a 3D-printed model. (F and G) Pre-bent plates and screws. (E) Originates from our previous study^[30]^ and was authorized by the Chinese Journal of Orthopaedics.

In the case group, the patients were required to undergo 1.0-mm CT (Aquilion 64, Toshiba, Tokyo, Japan) of the thin pelvic layer. The data were converted to DICOM images and uploaded to the medical image processing software (MIMICS, version 15, Belgium). A 3D image of the isolated malformed pelvis was created by subtracting the bilateral femurs using the splitting process (Fig. 1) and then exporting in STL format. Subsequently, the STL files were loaded onto a 3D printing machine (Objet30, Stratasys, America) using rapid prototyping technology to generate patient-specific pelvis 3D real models (1:1 model) (Fig. 2D). The osteotomy plane was also determined (Fig. 2A–D) and surgery guide was designed (Fig. 3) using MIMICS. The surgery guide was then manufactured by the 3D printing machine (Fig. 3). The simulated osteotomy was performed on the 3D model used as a surgery guide (Fig. 4A–C). The 3.5-mm reconstruction plate was contoured according to the reduction in the 3D model with bone transplantation simulated by a quantified module (Fig. 4D–G). The pre-contoured plates, screws, surgery guide, and bone graft module were disinfected for surgery.

**Figure 5.**
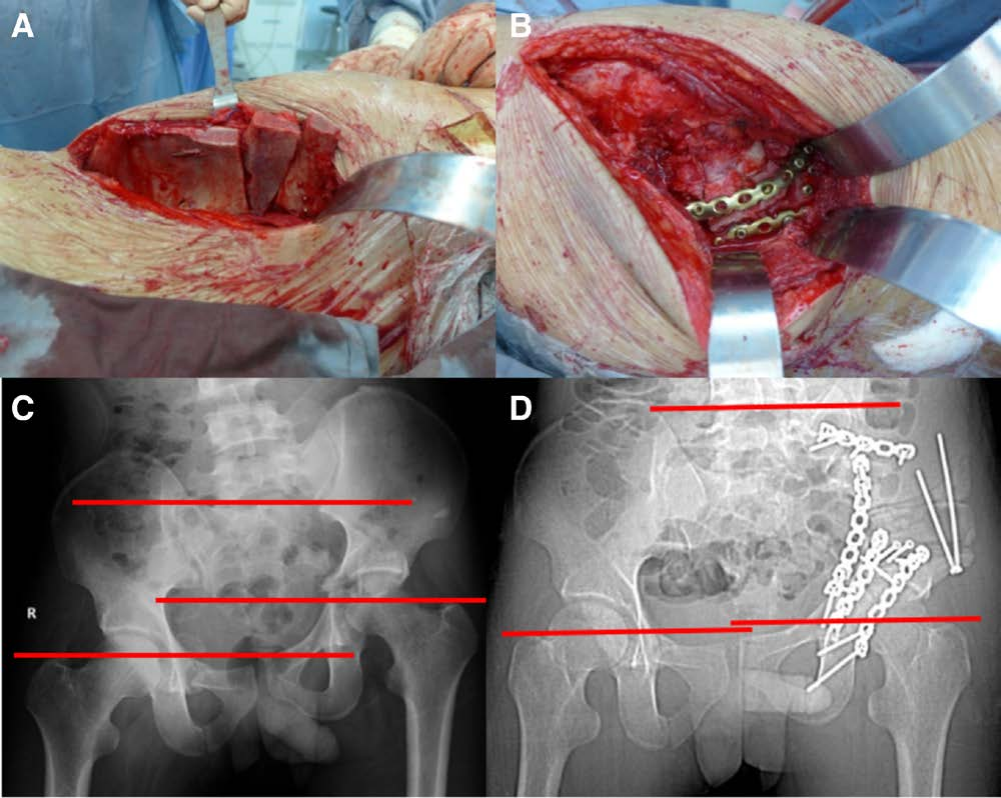
(A) Intraoperative bone graft after osteotomy. (B) Fixation with pre-bent plates. (C) Preoperative image showing limb discrepancy. (D) Postoperative radiography showing correction of limb discrepancy. (C and D) Originates from our previous study^[30]^ and was authorized by the Chinese Journal of Orthopaedics.

During the surgery, the ilioinguinal and/or Kocher–Langenbeck approaches were used based on osteotomy position. Osteotomy was performed based on the surgery guide. The transplanted bone block was from the iliac ala and molded in accordance with the quantified module. The bone grafts were fixed with 3.5-mm pre-contoured reconstruction plate (Fig. 5A–D). If acetabular fracture was present, the acetabulum was reconstructed and fixed with 3.5-mm pre-contoured reconstruction plate.

In the control group, the preoperative plan was implemented according to plain X-rays and CT scans. The osteotomy line was determined based on radiological results. After osteotomy and reduction, a 3.5-mm reconstruction plate was contoured with the assistance of a bending instrument intraoperatively.

### 2.2. Evaluation of the outcomes

Pelvic fracture types were categorized according to the Tile classification,^[11]^ and acetubular fractures were classified using the Judet–Letournel classification.^[12]^ The pelvic outcomes were evaluated at the final follow-up using the Majeed score.^[13]^ Hip joint function was evaluated based on the modified Merle D’Aubigné and Postel scoring system.^[14]^ Pain was evaluated according to visual analog scale (VAS). The residual displacement of the pelvis was graded using the method of Tornetta and Matta.^[15]^ The reduction in the acetabulum was assessed using the Matta system.^[16]^ Excellent and good results were considered satisfactory results. Radiographs were assessed by 2 experienced musculoskeletal radiologists who did not participate in the surgeries. Both observers achieved exact agreement in evaluating all images (weighted kappa was 1 for the pelvis and acetabulum).

### 2.3. Statistical analysis

Statistical analysis was conducted using SPSS version 16.0 for Windows (SPSS Inc., Chicago, IL). The results are presented as mean ± standard deviation. Continuous variables that were normally distributed between the groups were compared using the *t*-test. Fisher exact test was used for nominal data. Multiple logistic regression analysis was performed in order to identify variables that were independently associated with pelvic outcomes. A *P* value of .05 or less was considered to be statistically significant (two-tailed).

## 3. Results

### 3.1. Patients’ details

Fifty-five patients with inveterate pelvic and acetabular fractures were identified, and 18 patients were excluded. Thirty-seven patients were enrolled in this study. Seventeen patients were divided into the case group, and twenty patients were divided into the control group. Demographic information of these patients is listed in Table 1. The mean ages in the case and control groups were 36.0 ± 5.8 years (range, 27–52) and 37.4 ± 7.6 years (range, 25–57), respectively. Motor vehicle collision was the most common mechanism of injury in both groups. The times from injury to surgery were 111.1 ± 42.6 days (range, 45–230) in the case group and 121.4 ± 55.3 days (range, 37–279) in the control group.

**Table 1:** Demographic information.

Characteristics	Case group 17 patients	Control group 20 patients	*P* value
Age (years)	36.0 ± 5.8	37.4 ± 7.6	.551
*Sex* Male Female	12 5	14 6	.969
*Mechanism of injury* Motor vehicle collision Falling injury Crushing injury Duration from injury to surgery(days)	12 2 1 111.1 ± 42.6	12 3 2 121.4 ± 55.3	.795 .604

The fracture types are listed in Table 2. In the case group, 4 patients had Tile B fractures and 13 patients had Tile C fractures. Thirteen patients sustained acetabular fractures, according to the Judet–Letournel classification; 5 were elementary fractures, and 8 were associated fractures. Five patients had transverse fractures, 3 had anterior column with posterior half transverse fractures, 2 had fractures in both columns, 2 had a transverse with posterior wall fracture, and 1 had a posterior column with posterior wall fracture. In the control group, 5 patients had Tile B fractures and 15 patients had Tile C fractures. Fourteen patients sustained acetabular fractures; 3 were elementary fractures and 11 were associated fractures. Three patients had anterior column with posterior half transverse fractures, 1 had a T-type fracture, 2 had a fracture in both columns, 3 had transverse with posterior wall fractures, and 2 had posterior column with posterior wall fractures.

**Table 2:** Fracture types.

Characteristics	Case group	Control group
*Tile classification* A B C *Judet–Letournel classification* Elementary fracture Subtype Transverse Associated fracture *Subtype* Posterior column with posterior wall Transverse with posterior wall T-type Anterior column with posterior half transverse fracture Both column	0 4 13 5 5 8 1 2 0 3 2	0 5 15 3 3 11 2 3 1 3 2

Clinical data including age, sex, mechanism of injury, time from injury to operation, and fracture type were not significantly different between the 2 groups (*P* > .05, Table 1).

### 3.2. Peri-operative data

Peri-operative data are shown in Table 3. The average operation times in the case and control groups were 171.6 ± 27.9 min (range, 134–228) and 227.7 ± 42.0 min (range, 143–290), respectively. The difference was statistically significant (*P* < .001). The patients in the case group also experienced less blood loss with a volume of 1222.1 ± 374.9 mL (range, 650–1798) versus 1752.3 ± 521.5 mL (range, 1012–2710) in the control group (*P* = .004). Furthermore, there was a greater need for blood transfusion in the control group with 10.9 ± 3.4 units of packed red blood cells (range, 5–16), compared with 7.4 ± 2.3 units in the case group (range, 4–12) (*P* = .003). In the case group, the mean fluoroscopy time was 15.3 ± 5.1 s (range, 7–23), but in the control group, the time was significantly higher with an average of 22.2 ± 7.0 s (range, 13–35) (*P* < .002).

**Table 3:** Peri-operative data.

Characteristics	Case group 17 patients	Control group 20 patients	*P* value
Operation time Blood loss (mL), M ± SD Transfusion volume (units) Fluoroscopy time (s)	171.6 ± 27.9 1222.1 ± 374.9 7.4 ± 2.3 15.3 ± 5.1	227.7 ± 42.0 1752.3 ± 521.5 10.9 ± 3.4 22.2 ± 7.0	<.001 .004 .003 .002

### 3.3. Outcome evaluation

The average follow-up times in the case and control groups were 19.4 ± 4.6 months (range, 12–26) and 21.4 ± 5.3 months (range, 14–30), respectively. There was no significant difference in preoperative functional parameters including Majeed, Merle D’Aubigné and Postel, and VAS scores (*P* > .05, Table 4). After surgery, the average Majeed scores were 84.4 ± 3.8 in the case group and 81.4 ± 5.9 in the control group. The mean Merle D’Aubigné and Postel scores were 15.3 ± 1.4 in the case group and 14.9 ± 1.3 in the control group. Although improved function was noted after the surgery, no significant difference was shown in pelvic and hip joint outcomes (*P* > .05, Table 4). The VAS score improved from 6.3 ± 1.5 to 1.7 ± 1.1 in the case group and from 6.0 ± 1.4 to 2.8 ± 1.1 in the control group. Patients in the case group showed significantly lower pain grade at the last follow-up (*P* = .009, Table 4). To determine the influential factors of pelvic outcomes, the age, duration from injury to surgery, operation time, blood loss, transfusion volume, fracture type, and the application of 3D printing were assessed using univariable analysis and a multivariable logistic regression model, but no factor was found to be associated with pelvic outcomes.

**Table 4:** Outcome evaluation.

Result	Case group 17 patients	Control group 20 patients	*P* value
*Majeed score* Preoperative Postoperative *Merle D’Aubigné and Postel score* Preoperative Postoperative *VAS* Preoperative Postoperative *Matta radiological result (Pelvis*) Satisfactory Unsatisfactory *Matta radiological result (Acetabulum*) Satisfactory Unsatisfactory	70.9 ± 5.3 84.4 ± 3.8 12.1 ± 1.8 15.3 ± 1.4 6.3 ± 1.5 1.7 ± 1.1 12 5 10 3	69.7 ± 5.9 81.4 ± 5.9 12.4 ± 2.1 14.9 ± 1.3 6.0 ± 1.4 2.8 ± 1.1 11 9 8 6	.473 .204 .603 .32 .651 .009 .33 0.276

The radiological outcomes are shown in Table 4. In the case group, the radiological results were excellent in 7 patients, good in 5 patients, and fair in 5 patients. In the control group, the results were excellent in 5 patients, good in 6 patients, fair in 8 patients, and poor in 1 patient. The rate of satisfactory results was higher in the case group (70.59%) compared with the control group (55.00%), but the difference was insignificant (*P* = .33). The rate of satisfactory results of acetabular reduction was also higher in the case group compared with the control group (76.92% versus 57.14%). However, no significant difference was shown in either (*P* = .276).

### 3.4. Complications

Sciatic nerve injury occurred in 2 patients in the control group. One patient in each group had venous thrombosis in the lower limb. One patient developed femoral head necrosis which was in the control group, and one patient suffered hip osteoarthritis after surgery which was in the case group. However, all of them refused surgical treatment during the follow-up period. Nonunion or mechanical complications such as instrumentation failure did not occur.

## 4. Discussion

The development of 3D printing techniques provides an accurate 3D model of fracture and facilitates surgical simulation. A previous study suggested that 3D printing of a fracture model was of positive significance, as it could improve the preoperative plan and reduce difficulties for clinicians by shortening the learning curve.^[17]^ The segmented bones or fragments presented in a 3D model can be manipulated in a virtual simulation and therefore help better determine the reduction procedures.^[9]^ Three-dimensional printing techniques have been widely used in pelvic and acetabular surgeries, and surgeons have concluded that they can optimize surgeries, improve outcomes, and reduce complications.^[8–10,18–23]^

Surgeries for inveterate pelvic and acetabular fractures usually take long durations of an average of more than 6 hours and are associated with excessive bleeding.^[24]^ Three-dimensional models help improve the strategies of osteotomy and bone grafting and make it possible to prepare a pre-contoured plate and predefined number and length of screws by surgical simulation and reduce the surgical time.^[25]^ In this study, the average operation time in the case group was significantly less than that in the control group. The surgical simulation using 3D models may offer the surgeon a better understanding of the morphology preoperatively to then perform fracture reduction more easily.^[9]^ The decreased instrumentation time in the 3D printing group than in the traditional method group because of the utilization of pre-contoured plates is also a critical factor.^[9,26]^ Another advantage of 3D printing shown in our results was that the patients in the case group showed significantly reduced intraoperative blood loss and need of transfusion. The surgery is performed with smaller incisions and less soft tissue injury with the help of 3D printing models.^[9,27]^ The decreased operation time is in favor of reduced blood loss. Reduced operation time, blood loss, and fluoroscopy time have been reported in different studies concerning 3D printing models in pelvic, acetabular, joint, and upper limb fractures.^[7,9,23,28–30]^ Our results are consistent with previous findings, and we believe that the application of 3D models is in favor of a smoother execution of operations, making intraoperative reduction easier. The patients benefitted from surgical simulation with reduced blood loss, soft tissue injury, and radiation.

The patterns in inveterate pelvic and acetabular fractures differed significantly in different patients. The complexity of fractures and malunion with callus formation made it difficult to achieve anatomical reduction. Although a more intuitive understanding of the fracture morphology can be provided by 3D printing models, whether an improved quality of reduction can be achieved remains unclear. Some reports have suggested that improved reduction could be acquired for patients with pelvic and acetabular fractures with the help of 3D models.^[5,8,20]^ However, others declare that although a higher quality of reduction is observed in cases involving preoperative 3D printing plans compared with traditional surgery, the difference is insignificant.^[9,26,29]^ In our cases, according to the Matta radiological accession system, patients in the case group showed a better rate of satisfactory results for the pelvis (70.6%) and acetabulum (76.9%) compared with the control group (55% for the pelvis and 57.1% for the acetabulum), but there was no statistical significance between the 2 groups. The small sample size probably leads to the statistically insignificant result.

Inveterate pelvic and acetabular fractures usually have a course of more than 3 weeks.^[2]^ Indications for surgery include pelvic instability and clinical problems related to pelvic deformity. Most patients with inveterate pelvic fractures are in stable condition and visit the hospital because of unbearable symptoms. Common symptoms include pain, malformation, and abnormal gait. Function improvement is the main reason for secondary treatment. In our patients, both groups showed obvious improvement of pelvic and hip function after the surgery. However, no significant difference was shown between the 2 groups (*P* > .05). This insignificant function improvement has also been demonstrated in other studies.^[7,10,23,28,29]^ The compression of adjacent nerves caused by ectopic pelvis may also give rise to dysfunction of the bowel and urogenital system along with pelvic pain. Lindahl et al reported 39% of patients with pelvic malunion that presented with obvious pain in the pelvic region.^[29]^ Among our patients, the postoperative VAS pain score was significantly lower in the case group (*P* = .009). Given that there was no evident difference in function outcomes, we believe the better pain alleviation in the case group was in part due to the advantage of 3D printing model in the preoperative plan, which decreased surgical invasiveness and improved the accuracy of reduction. Our results demonstrated that patients who undergo 3D printing model-assisted preoperative planning show no significant functional improvement, but acquire better pain relief.

The decision to proceed with surgery should be made only after careful assessment in consideration of the reconstruction of pelvic malunion being associated with a high incidence of complications.^[24]^ In this study, venous thrombosis in the lower limb occurred in one patient in each group and was treated with anticoagulation therapy. Two patient experienced sciatic nerve injury in control group and was treated with neural nutrients. Complete recovery of this patient was observed at the 6-month follow-up after the surgery. No surgery-related neurovascular complications occurred in the case group. Previous studies have shown a reduced rate of postoperative complications following 3D model-aided surgery in patients with pelvic and acetabular fractures.^[7,9,27]^ Since surgical simulation make surgeries less invasive and significantly reduce the operation time, we believe that the usage of 3D printing techniques may reduce the rate of complications, but more cases are needed in future studies.

The application of 3D printing technology in orthopedics is widely reported. However, to our knowledge, this is the first study that focuses on its use in the treatment of inveterate pelvic and acetabular fractures. There are still several limitations in this study. This was a retrospective study, and the use of 3D printing mainly depended on patients’ willingness. The duration from injury to surgery differed greatly in patients from 37 to 279 days. The number of patients included in this study was relatively small, and a larger sample size is needed for a more exact conclusion of the impact of 3D printing techniques on clinical outcomes in a future study.

## 5. Conclusions

Compared with conventional radiographic planning, the application of 3D printing techniques can reduce the operative time and blood loss and lower the requirement of fluoroscopy. Patients who undergo 3D model-aided surgery gain better pain relief, but do not show significantly better function outcomes or higher quality of reduction. Our results demonstrated that for patients with inveterate pelvic and acetabular fractures, the usage of 3D printing techniques is of great importance in preoperative preparation and optimization of surgery.

## Author contributions

**Conceptualization:** Fulin Tao, Lin Li, Dawei Wang, Jinlei Dong, Dongsheng Zhou, Wenhao Song.

**Data curation:** Fulin Tao, Lin Li, Dawei Wang, Jinlei Dong, Dongsheng Zhou, Wenhao Song.

**Formal analysis:** Fulin Tao, Lin Li, Dawei Wang, Jinlei Dong, Dongsheng Zhou, Wenhao Song.

**Funding acquisition:** Dawei Wang, Dongsheng Zhou, Wenhao Song.

**Investigation:** Fulin Tao, Lin Li, Dawei Wang, Jinlei Dong, Dongsheng Zhou, Wenhao Song.

**Methodology:** Fulin Tao, Lin Li, Jinlei Dong, Dongsheng Zhou, Wenhao Song.

**Project administration:** Fulin Tao, Lin Li, Dawei Wang, Jinlei Dong, Wenhao Song.

**Resources:** Lin Li, Dawei Wang, Jinlei Dong, Dongsheng Zhou, Wenhao Song.

**Software:** Lin Li, Wenhao Song.

**Supervision:** Fulin Tao, Lin Li, Dawei Wang, Wenhao Song.

**Validation:** Fulin Tao, Lin Li, Jinlei Dong, Dongsheng Zhou, Wenhao Song.

**Visualization:** Fulin Tao, Lin Li, Dawei Wang, Jinlei Dong, Dongsheng Zhou, Wenhao Song.

**Writing – original draft:** Lin Li, Wenhao Song.

**Writing – review & editing:** Fulin Tao, Lin Li, Dawei Wang, Jinlei Dong, Dongsheng Zhou, Wenhao Song.
